# Determination of metal ion content of beverages and estimation of target hazard quotients: a comparative study

**DOI:** 10.1186/1752-153X-2-13

**Published:** 2008-06-25

**Authors:** Theresa Hague, Andrea Petroczi, Paul LR Andrews, James Barker, Declan P Naughton

**Affiliations:** 1 School of Life Sciences, Kingston University, Kingston upon Thames, Surrey KT1 2EE, UK; 2 Division of Basic Medical Sciences, St George's University of London, London SW17 0RE, UK; 3 School of Pharmacy and Chemistry, Kingston University, London KT1 2EE, UK

## Abstract

**Background:**

Considerable research has been directed towards the roles of metal ions in nutrition with metal ion toxicity attracting particular attention. The aim of this study is to measure the levels of metal ions found in selected beverages (red wine, stout and apple juice) and to determine their potential detrimental effects via calculation of the Target Hazard Quotients (THQ) for 250 mL daily consumption.

**Results:**

The levels (mean ± SEM) and diversity of metals determined by ICP-MS were highest for red wine samples (30 metals totalling 5620.54 ± 123.86 ppb) followed by apple juice (15 metals totalling 1339.87 ± 10.84 ppb) and stout (14 metals totalling 464.85 ± 46.74 ppb). The combined THQ values were determined based upon levels of V, Cr, Mn, Ni, Cu, Zn and Pb which gave red wine samples the highest value (5100.96 ± 118.93 ppb) followed by apple juice (666.44 ± 7.67 ppb) and stout (328.41 ± 42.36 ppb). The THQ values were as follows: apple juice (male 3.11, female 3.87), stout (male 1.84, female 2.19), red wine (male 126.52, female 157.22) and ultra-filtered red wine (male 110.48, female 137.29).

**Conclusion:**

This study reports relatively high levels of metal ions in red wine, which give a very high THQ value suggesting potential hazardous exposure over a lifetime for those who consume at least 250 mL daily. In addition to the known hazardous metals (e.g. Pb), many metals (e.g. Rb) have not had their biological effects systematically investigated and hence the impact of sustained ingestion is not known.

## Background

The widespread roles of metal ions in health and disease range from the requirement for intake of essential trace elements to toxicity associated with metal overload. Numerous studies have quantified levels of toxic metal ions in common foodstuffs and in some cases these results have been extended by statistical analyses to generate 'target hazard quotients' (THQ) for a limited number of foodstuffs relating to individual and combinations of metals [1-5]. Despite regulatory controls, numerous sources of metal ingestion have been recently reported including contaminated drinking water, seafood, breast milk, herbal medicines, smoking, together with plants and animals used in the diet [1-10]. Between January and November 2007, some 2,680 food recalls were monitored by the Laboratory of the Government Chemist (UK) mainly owing to contamination [11]. Of these, 8% (216) were for metal contamination with 79% (171) of these recalls being for seafood with the rank order of metals involved being Hg (58%), Cd (26%), Pb (10.5%), As (3.5%), Ba (<1%) and Zn (<1%). Serious incidents of occasional human exposure to metals have resulted from lapses in quality control measures or errors in processing [11].

In addition to the endogenous metal ion content of foodstuffs, numerous steps during processing and packaging may add to the metal ion load. This is exemplified by the authorised use of the many metal containing additives such as the 'antioxidant' stannous chloride (E512). Perhaps, the constant exposure that accompanies repetitive ingestion of foodstuffs that contain known low levels of metal ions is of more consequence. A key potential source of exposure is processed beverages that are known to contain metals, and frequent exposure may result in accumulative effects. Common beverages are assessed for their metal content with regulatory controls over maximum permitted levels in place in most countries. These permitted levels are subject to frequent review and revisions should take account of varying dietary habits in different populations and countries. The metal ion content in beverages has been determined in several studies for consumer protection against contamination, method development or an evaluation of the nutritional status [12-15]. The metal profiles have been reportedly used for the determination of the region of origin [16,17]. The majority of studies report levels of metal ions below the regulatory safe limits for that region. However, studies rarely report the combined effects of frequent ingestion of multiple metal ions which can be addressed as a function of the quantified level of concern in the form of THQ values arising from individual or combined metal concentrations. No studies have reported the individual and combined THQ values for common beverages.

The extension of lifespan in the Western world, in addition to dietary exposure accounting for the largest exposure to metals, warrants regular reviews of the total potential accumulation load over a lifetime. Many metal ions are associated with enhanced oxidative stress, inflammation and cancer [18,19]. The aim of this paper is to assess the levels of metal ions in three processed, commercially-available beverages. In total, the levels of thirty metal ions have been assessed by inductively coupled plasma-mass spectrometry (ICP-MS) for intact red wine, stout and apple juice which were selected as representative processed beverages arising from plants. In order to estimate the levels of metals that are non-protein bound, the levels in the beverage with highest levels (red wine) have been assessed in both the intact fluid and after ultra-filtration. These data were used to calculate the THQ values for key potentially toxic metal ions separately for females and males.

## Results

### Metal contents of beverages

**Figure 1:**
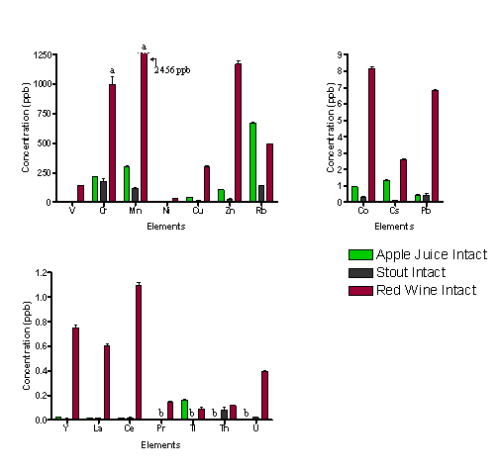
**ICP-MS results for intact beverages in the concentration ranges of high (up to 1250 ppb); medium (up to 9 ppb); low (up to 1.5 ppb)**. a = above working range.

**Figure 2:**
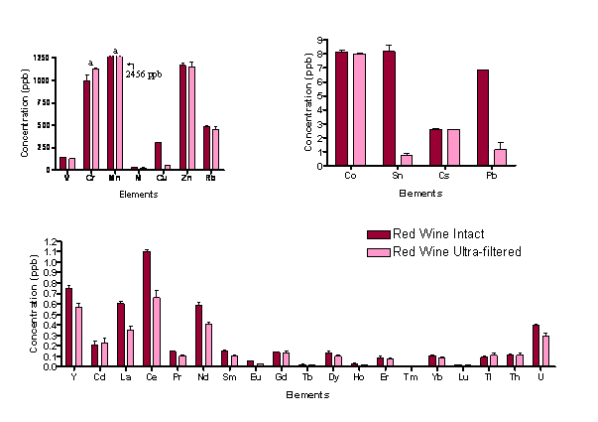
**ICP-MS results for intact and ultra-filtered red wine in the concentration ranges of high (up to 1250 ppb); medium (up to 9 ppb); low (up to 1.5 ppb)**. a = above working range.

The concentrations of thirty metal ions assessed by ICP-MS are shown in Figures 1 and 2 (for exact values, see additional file 1: Quantification of metal ions in whole apple juice and stout and additional file 2: Quantification of metal ions in red wine pre- and post- ultra-filtration) for red wine, stout and apple juice in parts per billion with the corresponding standard errors of the mean (SEM) values. Concentrations are presented in Figures 1 and 2 in three ranges for convenience: high (up to 1,250 ppb); medium (up to 9 ppb); low (up to 1.5 ppb). Wide variations in the types and levels of metals were detected in the beverages, ranging from some fifteen metals in the apple juice and stout, to thirty in the red wine sample (see additional file 1: Quantification of metal ions in whole apple juice and stout and additional file 2: Quantification of metal ions in red wine pre- and post- ultra-filtration) based on the lower limits of detection (see additional file 2: Quantification of metal ions in red wine pre- and post- ultra-filtration). Figure 1 clearly shows that intact red wine contains the highest levels and diversity of metals which includes several metals that are not detected in the other beverages (e.g. V, Ni, Sn, Cd, and a range of lanthanides). In contrast, many metals were detected in all three beverages (e.g. Cr, Rb, Co, Pb).

In the apple juice samples, the predominant metals are, in order of concentration, Rb>Mn>Cr>Zn>Cu, with a total measured metal ion concentration of 1,339.87 ± 10.84 ppb. For stout the total metal concentration was 464.86 ± 46.74 ppb in the order of Cr>Rb>Mn>Zn>Cu.

The total metal ion content measured for the 30 elements was 5,620.54 ± 123.86 ppb for red wine, with the order being Mn>Cr>Zn>Rb>Cu>V for the elements found at the highest concentrations. Red wine contained the most varied composition of metal ions which were also in the highest quantities (Figure 1 and see additional file 2: Quantification of metal ions in red wine, pre- and post- ultra-filtration). Owing to the high levels of potentially hazardous metal ions, for these analyses, the results were recorded for both the intact beverages and the filtrates after ultra-filtration to remove the components above a molecular weight of 5,000 (nominal). In red wine, a considerable proportion of the metal ion content exists after ultrafiltration. Fourteen metals had lower percentage levels in the ultrafiltrate that were statistically significant (see additional file 2: Quantification of metal ions in red wine, pre- and post- ultra-filtration). Of these, nine were approaching 50% in the low molecular mass fraction, with only V in the highest concentration range, the rest being in the lowest concentration range. Thus, quite high levels of metal ions exist in the both the intact and low molecular mass portions except for Cu.

For the intact samples, statistically significant (p < 0.05) differences were found between the metal contents of the apple juice, stout and red wine for all metals except Th (p = 0.137). Differences in metal ion concentration were calculated from the data (see additional file 1: Quantification of metal ions in whole apple juice and stout, additional file 2: Quantification of metal ions in red wine pre- and post- ultra-filtration), and these significant differences are highlighted in Table 1. The total levels of metal ions selected for assessment of the THQ values were: red wine 5,107.76 ± 118.93 ppb, apple juice 666.44 ± 7.67 ppb and stout 328.41 ± 42.35 ppb.

**Table 1:** Summary of Tukey HSD post hoc comparisons (p < 0.05) of apple juice, stout and red wine

Differences	Elements
In all pairs (i.e. Red wine ↔ Apple juice, Red wine ↔ Stout, Apple juice ↔ Stout)	Cs, Co, Cu, Mn, Rb, Tl, Zn
Red wine ↔ (Apple juice and Stout)	Ce, Cr, Dy, Er, Eu, Gd, Ho, La, Lu, Ni, Pr, Nd, Pb, Sm, Sn, Tb, Tm, U, V, Y, Yb
Stout ↔ (Apple juice and Red wine)	Cd

↔ denotes difference

## Discussion

### Target hazard quotients

The THQ is calculated by the formula established by the Environmental Protection Agency [20] using equation 1, where EFr is the exposure frequency (days/year); ED_tot _is the exposure duration (year); SFI is the mass of selected dietary ingested (g/day); MCS_inorg _is the concentration of inorganic species in the dietary components (μg/g wet weight); R_f_D: oral reference dose (mg/kg/day); BW_a_: the average adult body weight; AT_n_: averaging time for non-carcinogens (day); and 10^-3^: the unit conversion factor.

(1) $$THQ=\frac{EFr\times ED_{tot}\times SFI\times MCS_{inorg}}{R_{f}D\times BW_{a}\times AT_{n}}\times 10^{-3}$$

The interpretation of the THQ value is binary: THQ is either ≥ 1 or < 1, where THQ > 1 indicates a reason for health concern [20]. It must be noted that THQ is not a measure of risk [21] but indicates a level of concern and while the THQ values are additive, they are not multiplicative: e.g. the level of concern at THQ of 20 is larger but not tenfold of those at THQ = 2.

**Figure 3:**
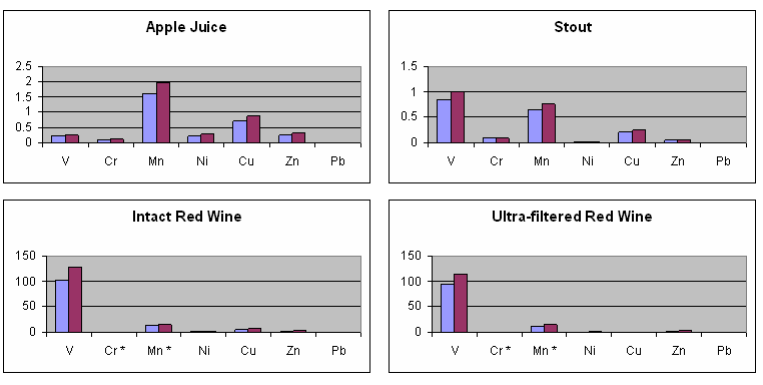
**Individual Target Hazard Quotients for all beverages for males (blue) and females (red)**. * above working range.

**Figure 4:**
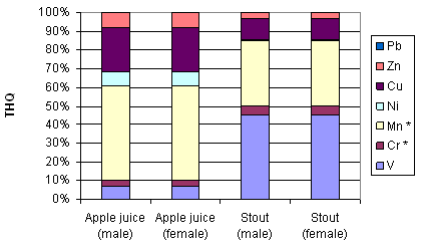
Combined Target Hazard Quotients for apple juice and stout expressed as percentages.

Individual THQ values approach or surpass 1 for two metals in stout (V, Mn) and apple juice (Cu, Mn) (Figures 3 and 4). The contributions from these metals bring the combined THQ values to approximately 2.00 and 3.49 for stout and apple juice, respectively. Owing to differences in average weight and lifespan, the THQ values are raised for females.

**Figure 5:**
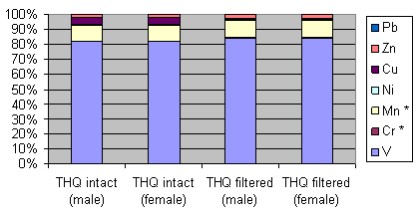
**Combined Target Hazard Quotients for red wine**. * above working range.

In contrast, the THQ values for individual elements in red wine are dominated by V (>100) with high values for Mn (>10), Cu (>5), Zn (>2.8) and Ni (>1). The combined THQ values in the case of red wine exceed 125. As expected, the levels for females are raised. When expressed as a percentage, V contributes over 80% with Mn affording some 10% to the total THQ for red wine (Figure 5). With ultrafiltration, the THQ values reduce slightly for all elements, owing to removal of high molecular mass species, except for Cu which exhibits a significant decrease (see additional file 3: THQ values for intact beverages and ultra-filtered red wine).

Many of the toxic effects associated with metals are still under investigation, especially for low concentrations and for lifetime exposure. It is notable that for many metal ions, upper safe limits are unavailable which prevents THQ estimations [22]. Apart from some well recognised cases of metal ion overload, the full effects of metal ions in the body may remain in the realm of sub-clinical pathology acting through numerous mechanisms including oxidative stress. In red wine, a small proportion of the metals are in the high molecular mass form and are probably bound to, or encapsulated in, macromolecules such as proteins. For the detailed discussion of the metals contributing to the high THQ value, particularly in red wine, it must be noted that individual differences such as a persons' genetic make up, environment or co-exposures were not considered.

### Vanadium

Although it is poorly absorbed in the gastrointestinal (GI) tract (ca 2%), V has numerous reported toxic effects which include the liver, kidney, nervous system, cardiovascular system, and blood-forming organs along with an ability to target mitochondria and mediate oxidative stress [23,24]. In the absence of a proven beneficial role in humans along with a paucity of information on the toxic effects, an upper safe limit for V is difficult to substantiate. However, for the purposes of this study we have adopted those commonly used in this field [2,25,26]. THQ values for this element alone of ca 1.0 in stout are notable but the THQ values of >100 in red wine are of particular concern, especially as some 50% of this is as low molecular weight species.

### Manganese

In contrast to V, Mn has numerous uses in the body being a component of several enzymes (e.g. superoxide dismutase). It is found in a number of food stuffs including bread, tea and drinking water. Commonly found in supplements, further studies on this cumulative neurotoxin are required to authoritatively state the upper safe limit. The contribution of Mn to the THQ values for all three beverages is similar as expected for this ubiquitous element indicating that relatively high doses are ingested in a normal diet.

### Chromium

Chromium toxicity, like mercury, is very dependent on the species and oxidation states present and is still subject to considerable debate [27-29]. It is normally found in the considerably less toxic trivalent state in foodstuffs, is poorly absorbed in the GI tract and is reported to have beneficial effects on type II diabetes. The hexavalent toxic form participates in oxidative stress [29]. The uncertainties that surround the mechanisms of chromium toxicity, along with its ability to potentiate oxidative stress, warrant concern regarding the levels of this element in red wine, particularly in the low molecular mass form.

### Zinc and copper

Zinc and copper have numerous reported beneficial and detrimental effects, being essential components of many enzymes [30,31]. The interacting intestinal uptake mechanisms can lead to imbalances in absorption of copper which is proposed as a major manifestation of zinc toxicity. Other reported effects include decreased levels of superoxide dismutase, a key enzyme for protection against oxidative damage which may be enhanced by labile copper, which may initiate or exacerbate inflammatory disorders [18]. Copper toxicity is rarely encountered outside of specific disorders, although high levels may prevent Zn uptake. Although it exists predominantly in the high molecular mass form in red wine, release during protein degradation enhances the redox active metal ion burden as a part of the THQ.

### Nickel and Lead

Nickel levels made a moderate contribution to the combined THQ in red wine. Nickel has numerous reported mechanisms of toxicity including redox-cycling and inhibition of DNA repair as well as exhibiting allergenic/sensitising effects. The toxic effects of lead are well documented, particularly as a neurotoxin [32]. Lead is commonly found in foodstuffs such as tea with a recent report of 32% of 1,225 Chinese tea samples exceeding the upper limit for lead [33]. Only the levels found in red wine are significant but do not reach the level of concern in terms of THQ values over the lifespan.

## Conclusion

This study is the first to assess the levels of metal ion exposure over a lifetime in terms of the THQ values for the common beverages stout, apple juice and red wine. The levels of metals found in red wine are of particular concern as they lead to very high THQ values and the ultra-filtrates contained approximately 50% of each metal ion. In addition to the known hazards of metal ion intake, it is noteworthy that a large number of metal ions found in these beverages, especially wine, have not been well studied in terms of biological activity. This approach should be extended to the numerous dietary products that are consumed daily over a lifetime.

The quantified level of concern expressed as THQ values may be expanded beyond the arbitrarily selected group of 250 mL per day ingestion by using an estimated consumption distribution in a given population and the probability of the metal content in selected foodstuffs. In order to translate the level of concern arising from the environment into potential risks to human health, modifying factors that may enhance or prohibit the body's ability to cope with metal exposure should also be taken into consideration.

## Experimental

Samples of one brand for each of the three beverages: apple juice (n = 3), stout (n = 4) and red wine (n = 4) were purchased from a local supermarket. The apple juice was pressed in the UK and contained in plastic bottles. The stout was 'foreign extra stout' in glass bottles from Ireland. The red wine was a Shiraz from South Eastern Australia contained in a screw cap glass bottle. All samples were diluted one in ten prior to analyses in triplicate by ICP-MS on a Quadrupole ICP-MS (Agilent Technologies 7500c) using a cross flow nebuliser. For red wine samples, two mL were placed by pipette into an ultrafiltration device (nominal molecular weight cut-off = 5,000) and centrifuged at 2,060 g for 20 mins. Results are presented in parts per billion '*ppb' *(μg/L) with standard errors of the mean (SEM).

The concentrations of the thirty metal ions in the intact samples were compared between the three beverages using One-way ANOVA test, followed by Tukey HSD post hoc comparisons if significant omnibus F was obtained. Statistically significant differences between the intact and filtered red wine samples were tested using paired sample t-test analysis. Owing to the small sample size, the level of significance was set to t/F > CV at 0.05.

To assess the level of concern arising from the metal concentrations, THQ values were calculated using the measured metal concentration in the intact samples for seven key potentially toxic metal ions for the three beverages and the ultra-filtered red wine. THQ is a ratio between the measured concentration and the oral reference dose, weighted by the length and frequency of exposure, amount ingested and body weight. The THQ values for selected metals were calculated using the method described previously [4] with the following oral reference doses in mg/kg/d [2,13]: V (1.0 × 10^-3^), Cr (1.5), Mn (1.4 × 10^-3^), Ni (2.0 × 10^-3^), Cu (4.0 × 10^-2^), Zn (3.0 × 10^-1^) and Pb (1.5). For this project, length of exposure was set to 17,155 days for males and for females based on the average life expectancy of 81.9 and 84.7, respectively from 18 years of age [34]; one large wine glass (250 mL) consumed daily for all three beverages. THQ was calculated separately for males and females using the mean life expectancies (71.9 and 84.7 years respectively) and the mean weight (83.11 and 69.81 kg respectively) [35]. For the oral reference dose we used the tolerable upper intake level (UL) [25,26], which is the highest average daily intake level without the risk of adverse health effects. Intake above the UL could be hazardous to health to almost all individuals in the general population.

## Competing interests

The authors declare that they have no competing interests.

## Authors' contributions

TH participated in the study design, collection of data, interpretation of results and preparation of the paper, AP participated in the study design, performed the statistical analyses and contributed to drafting the manuscript, PLRA participated in the study design, interpretation of results and preparation of the paper, JB participated in the study design, interpretation of results and preparation of the paper, DN participated in the study design, interpretation of results and preparation of the paper. All authors read and approved the final manuscript.

## Supplementary Material

Additional File 1 Quantification of metal ions in whole apple juice and stout. This file contains ICP-MS measurement data for apple juice and stout. Click here for file

Additional File 2 Quantification of metal ions in red wine pre- and post- ultra-filtration. This file contains ICP-MS measurement data for red wine. Click here for file

Additional File 3 THQ values for intact beverages and ultra-filtered red wine. This file contains individual and total THQ values for red wine. Click here for file
