# Supplementary material for: Determination of metal ion content of beverages and estimation of target hazard quotients: a comparative study
Source: Chem Cent J. 2008 Jun 25;2:13. doi: 10.1186/1752-153X-2-13 (PMC2443149; doi:10.1186/1752-153X-2-13)
Supplement: Additional File 1 — Quantification of metal ions in whole apple juice and stout. This file contains ICP-MS measurement data for apple juice and stout. [file 1752-153X-2-13-S1.doc]

**ICP-MS measurements of metal ions in whole apple juice and stout**

| Metal | Apple Juice (ppb) | Stout (ppb) |
| --- | --- | --- |
| V | 0.287 ± 0.038 | 1.091 ± 0.122 |
| Cr | 214.440 ± 0.732 | 173.946 ± 22.870 |
| Mn | 301.720 ± 2.504 | 116.480 ± 3.858 |
| Co | 0.942 ± 0.022 | 0.313 ± 0.026 |
| Ni | 6.425 ± 0.073 | 3.505 ± 0.526 |
| Cu | 39.065 ± 2.396 | 10.687 ± 1.430 |
| Zn | 104.088 ± 1.879 | 22.289 ± 13.437 |
| Rb | 670.948 ± 3.105 | 135.861 ± 4.313 |
| Y | 0.025 ± 0.000 | 0.011 ± 0.002 |
| Cs | 1.321 ± 0.033 | 0.127 ± 0.001 |
| La | 0.014 ± 0.001 | 0.014 ± 0.004 |
| Ce | 0.017 ± 0.000 | 0.017 ± 0.004 |
| Pr | 0.001 ± 0.001 | b |
| Tl | 0.162 ± 0.004 | b |
| Pb | (n) 0.418 ± 0.052 | 0.410 ± 0.112 |
| Th | 0.016 ± 0.003 | 0.083 ± 0.026 |
| U | b | 0.020 ± 0.005 |

Values are expressed as mean±SEM

The additional metal ions listed in additional file 2, for red wine, were not detected in apple juice and stout.

a = above working range

b = below level of detection

(n) near level of detection
