# Supplementary material for: Determination of metal ion content of beverages and estimation of target hazard quotients: a comparative study
Source: Chem Cent J. 2008 Jun 25;2:13. doi: 10.1186/1752-153X-2-13 (PMC2443149; doi:10.1186/1752-153X-2-13)
Supplement: Additional File 2 — Quantification of metal ions in red wine pre- and post- ultra-filtration. This file contains ICP-MS measurement data for red wine. [file 1752-153X-2-13-S2.doc]

**ICP-MS results for red wine pre- and post- ultra-filtration**

| Metal | LOD | Red Wine (ppb) | RW / LMW % | t | p |
| --- | --- | --- | --- | --- | --- |
| V | 0.00188 | 140.825 ± 1.141 | *89.89 | -4.860 | 0.017 |
| Cr^b^ | 0.02483 | 999.194 ± 64.180 | 112.99 | 1.978 | 0.142 |
| Mn^b^ | 0.00580 | 2456.137 ± 18.525 | 97.05 | -4.130 | 0.026 |
| Co | 0.00186 | 8.161 ± 0.105 | 98.02 | -1.811 | 0.168 |
| Ni | 0.01069 | 30.980 ± 3.972 | 85.07 | -1.015 | 0.385 |
| Cu | 0.03304 | 303.246 ± 3.322 | **16.53 | -30.946 | 0.000 |
| Zn | 0.80155 | 1170.561 ± 27.716 | 98.34 | -0.378 | 0.731 |
| Rb | 0.00259 | 489.084 ± 4.095 | 93.04 | -0.962 | 0.407 |
| Y | 0.00062 | 0.752 ± 0.023 | **76.12 | -9.585 | 0.002 |
| Cd | 0.01508 | (n) 0.206 ± 0.036 | 111.05 | 0.481 | 0.664 |
| Sn | 0.03274 | 8.193 ± 0.473 | **8.61 | -16.400 | 0.000 |
| Cs | 0.00133 | 2.593 ± 0.056 | 99.88 | -0.040 | 0.971 |
| La | 0.00083 | 0.605 ± 0.018 | *57.66 | -5.299 | 0.013 |
| Ce | 0.00053 | 1.099 ± 0.020 | **60.02 | -5.944 | 0.010 |
| Pr | 0.00059 | 0.147 ± 0.005 | *69.88 | -5.480 | 0.012 |
| Nd | 0.00290 | 0.585 ± 0.027 | *69.31 | -3.647 | 0.036 |
| Sm | 0.00338 | 0.150 ± 0.006 | **70.26 | -5.941 | 0.010 |
| Eu | 0.00065 | 0.056 ± 0.005 | **43.48 | -31.936 | 0.000 |
| Gd | 0.00700 | 0.139 ± 0.005 | 91.90 | -0.445 | 0.686 |
| Tb | 0.00031 | (n) 0.023 ± 0.001 | 71.53 | -2.815 | 0.067 |
| Dy | 0.00132 | 0.134 ± 0.013 | 75.77 | -2.878 | 0.064 |
| Ho | 0.00075 | 0.031 ± 0.004 | *67.02 | -4.574 | 0.020 |
| Er | 0.00099 | 0.089 ± 0.011 | 83.37 | -0.979 | 0.400 |
| Tm | 0.00079 | 0.010 ± 0.001 | 102.59 | 0.102 | 0.925 |
| Yb | 0.00357 | 0.104 ± 0.008 | *82.95 | -2.345 | 0.101 |
| Lu | 0.00064 | 0.017 ± 0.003 | 82.24 | -0.839 | 0.463 |
| Tl | 0.00452 | (n) 0.093 ± 0.008 | 119.97 | 0.733 | 0.517 |
| Pb | 0.01339 | 6.811 ± 0.073 | 16.55 | -10.415 | 0.002 |
| Th | 0.00075 | 0.118 ± 0.003 | *92.31 | -0.448 | 0.684 |
| U | 0.00092 | 0.396 ± 0.008 | *74.72 | -3.537 | 0.038 |

a = RW= red wine: LMW = low molecular weight fraction

b = above working range

(n) near LOD

G & RW Critical values = 3.182

* p ≤ 0.05 ** p ≤ 0.01 (t > CV = 4.303)
