# Supplementary material for: Determination of metal ion content of beverages and estimation of target hazard quotients: a comparative study
Source: Chem Cent J. 2008 Jun 25;2:13. doi: 10.1186/1752-153X-2-13 (PMC2443149; doi:10.1186/1752-153X-2-13)
Supplement: Additional File 3 — THQ values for intact beverages and ultra-filtered red wine. This file contains individual and total THQ values for red wine. [file 1752-153X-2-13-S3.doc]

**THQ values (individual and total) for intact beverages and ultra-filtered red wine**

| **Metal** | **Apple juice (male)** | **Apple juice (female)** | **Stout (male)** | **Stout (female)** | **Wine intact (male)** | **Wine intact (female)** | **Wine filtered (male)** | **Wine filtered (female)** |
| --- | --- | --- | --- | --- | --- | --- | --- | --- |
| V | 0.21 | 0.26 | 0.84 | 1.00 | 103.58 | 128.72 | 93.11 | 115.70 |
| Cr * | 0.11 | 0.13 | 0.09 | 0.11 | 0.49 | 0.61 | 0.55 | 0.69 |
| Mn * | 1.58 | 1.97 | 0.64 | 0.76 | 12.90 | 16.04 | 12.52 | 15.56 |
| Ni | 0.24 | 0.30 | 0.01 | 0.02 | 1.14 | 1.42 | 0.97 | 1.20 |
| Cu | 0.72 | 0.90 | 0.21 | 0.24 | 5.58 | 6.93 | 0.43 | 0.54 |
| Zn | 0.26 | 0.32 | 0.06 | 0.07 | 2.82 | 3.51 | 2.89 | 3.59 |
| Pb | 0.00 | 0.00 | 0.00 | 0.00 | 0.00 | 0.00 | 0.00 | 0.00 |
| Total | 3.11 | 3.87 | 1.84 | 2.19 | 126.52 | 157.22 | 110.48 | 137.29 |

***** above working range

.
